# Antidiabetic Components Contained in Vegetables and Legumes 

**DOI:** 10.3390/molecules13051189

**Published:** 2008-05-23

**Authors:** Guang-Yan Tang, Xue-Juan Li, Hong-Yu Zhang

**Affiliations:** School of Life Sciences, Shandong University of Technology, Zibo 255049, P. R. China

**Keywords:** Type 2 diabetes, vegetables, legumes, antidiabetic components, medicinal database

## Abstract

Epidemiological analyses in a large Chinese population have revealed that consumption of vegetables and legumes is inversely associated with the risk of type 2 diabetes (T2D). However, the health benefits of these plants have not been fully explained, which stimulated our interest to identify antidiabetic components from vegetables and legumes through searching medicinal databases, especially those containing traditional Chinese medicines. The results not only provide meaningful clues to understanding the antidiabetic potentials of these plants but also display the possibility of pinpointing food component functions by searching medicinal databases.

## Introduction

It is well known that certain foods may have the potential to prevent diseases [1,2]. For instance, the Mediterranean diet is helpful to lowering the risks of coronary heart disease, cancer and cognitive impairment [3,4,5]. Consumption of green tea is beneficial for preventing cancer and Alzheimer’s disease (AD) [6,7,8]. Recently, Villegas and co-workers reported that adherence to vegetables (including cruciferous vegetables, green leafy vegetables, yellow vegetables, allium vegetables, tomatoes and others) and legumes (including soybean, peanut, *etc.*) is inversely associated with the risk of type 2 diabetes (T2D) in a large Chinese population [9,10]. However, the health benefits of these plants have not been fully explained, which stimulated our interest to address this issue further. Considering the fact that some foods have been recognized as natural medicines, in particular some vegetables and legumes have been used as traditional medicines in China for many years, we speculated that it is highly possible to pinpoint food component functions by searching medicinal databases, especially those containing traditional Chinese medicines. 

**Figure 1:**
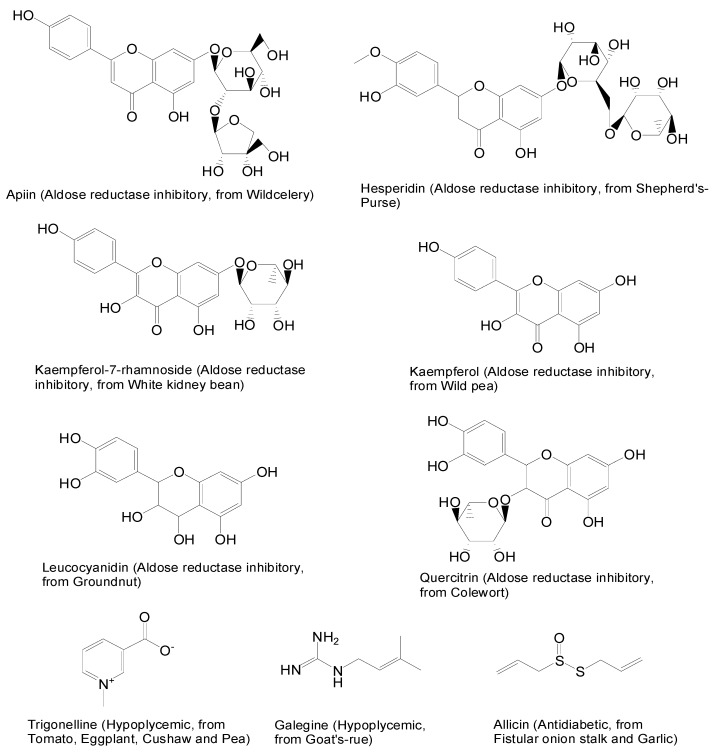
TCMD-documented vegetable and legume components with aldose reductase inhibitory or hypoplycemic activity.

## Results and Discussion

Primarily, we searched the Traditional Chinese Medicine Database (TCMD), which documents ~10,000 components extracted from ~4,600 traditional medicinal agents [11]. Hundreds of components were identified from vegetables and legumes that are recorded in the TCMD. According to the pharmacological activity annotations, we found that some components (Figure 1) are directly associated with prevention and/or treatment of T2D, because of their aldose reductase inhibitory or hypoplycemic activity. 

Besides, the functions of many other vegetable and legume components (*e.g.*, antiatherosclerotic, antihypertensive, antilipemic, antithrombotic, lipase inhibitory, lipid peroxidation inhibitory, lipoxygenase inhibitory and platelet aggregation inhibitory) are also associated with ameliorating T2D (Table 1) [12,13,14,15,16].

**Table 1:** TCMD-documented vegetable and legume functional components associated with ameliorating type 2 diabetes.

Compound	Activity	Source
Aframodial	Antilipemic	Zingiber (*Zingiber officinale Rosc.*)
Agavasaponin C	Platelet aggregation inhibitory	Garlic (*Allium sativum L.*)
Allicin	Antihypertensive;	Shallot (*Allium fislulosum L.*);
	Antithrombotic	Garlic (*Allium sativum L.*)
Alliin	Antithrombotic;	Onion (*Allium cepa L.*);
	Platelet aggregation inhibitory	Garlic (*Allium sativum L.*)
Bergapten	Antihypertensive	Tomato (*Lycopersicon esculentum Miller*)
beta-Sitosterol	Antilipemic	Black soybean (*Glycine max (L.) Merr.*)
Camphene	Antilipemic	Zingiber (*Zingiber officinale Rosc.*); Mint (*Mentha haplocalyx Briq.*)
Daidzein	Lipase inhibitory	Black soybean (*Glycine max (L.) Merr.*)
Ferulic acid	Platelet aggregation inhibitory	Onion (*Allium cepa L.*)
Genistein	Lipase inhibitory	Black soybean (*Glycine max (L.) Merr.*)
Glycitein	Lipoxygenase inhibitory	Black soybean (*Glycine max (L.) Merr.*)
Isoeruboside B	Platelet aggregation inhibitory	Garlic (*Allium sativum L.*)
Isorhamnetin	Antilipemic; Platelet aggregation inhibitory	Cress (*Oenanthe javanica (B1.)DC.*)
Kaempferol	Δ-5-lipoxygenase inhibitory	Wild pea (*Vicia amoena Fisch. ex DC.*)
Leucocyanidin	Platelet aggregation inhibitory	Groundnut (*Arachis hypogaea L.*)
Lycopene	Antiatherosclerotic	Tomato (*Lycopersicon esculentum Miller*); Bitter gourd (*Momordica charantia L.*)
Methyl allyl trisulfide	Platelet aggregation inhibitory	Garlic (*Allium sativum L.*)
Myristicin	Platelet aggregation inhibitory	Wild celery (*Apium graveolens L.*)
p-Coumaric acid	Antilipemic	Potato (*Solanum tuberosum L.*)
Proto-iso-eruboside B	Antithrombotic	Garlic (*Allium sativum L.*)
Rosmarinic acid	Antithrombotic; Platelet aggregation inhibitory	Mint (*Mentha haplocalyx Briq.*)
6-Shogaol	Antihypertensive; Platelet aggregation inhibitory	Zingiber (*Zingiber officinale Rosc.*)
Solasonine	Platelet aggregation inhibitory	Capsicum (*Capsicum annuum L.*); Eggplant (*Solanum melongena L.*)
Soyasaponin A1	Antilipemic; Antithrombotic	Black soybean (*Glycine max (L.) Merr.*)
Soyasaponin A2	Antilipemic	Black soybean (*Glycine max (L.) Merr.*)
Soyasaponin A3	Lipoxygenase inhibitory	Black soybean (*Glycine max (L.) Merr.*)
Soyasaponin A4	Lipoxygenase inhibitory	Black soybean (*Glycine max (L.) Merr.*)
Soyasaponin A5	Lipoxygenase inhibitory	Black soybean (*Glycine max (L.) Merr.*)
Soyasaponin A6	Lipoxygenase inhibitory	Black soybean (*Glycine max (L.) Merr.*)
Soyasaponin V	Lipoxygenase inhibitory	Black soybean (*Glycine max (L.) Merr.*); White kidney bean (*Phaseolus vulgaris L.*)
Stigmasterol	Antilipemic	Black soybean (*Glycine max (L.) Merr.*); Purple haricot (*Lablab purpureus (L.)Sweet*); Groundnut (*Arachis hypogaea L.*); White kidney bean (*Phaseolus vulgaris L.*)
Tomatine	Antihypertensive	Tomato (*Lycopersicon esculentum Miller*)
2-Vinyl-4H-1,3-dithiin	Platelet aggregation inhibitory; Antithrombotic; 5-lipoxygenase inhibitory	Garlic (*Allium sativum L.*)

Furthermore, through comparing the structures of these components with those recorded in the Comprehensive Medicinal Chemistry (CMC) database (which records ~8,000 clinically used drugs) [17] and the MDL Drug Data Report (MDDR) database (which collects ~145,000 drug candidates) [18], we found that some of these agents have been recognized by modern Western medicine (Table 2). Although some activities annotated in CMC and MDDR are not the same as displayed in TCMD, they are also associated with combating T2D. Taken together, the present analysis clearly indicates that vegetables and legumes indeed contain many antidiabetic components, which provide new clues to understanding the beneficial effects of vegetable and legume consumption on the risk of T2D [9,10]. 

**Table 2:** CMC- and MDDR-documented vegetable and legume functional components associated with ameliorating type 2 diabetes.

Compound	Activity
Allicin	Hypolipidemic (CMC/MDDR); Hypocholesterolemic (CMC); Platelet aggregation inhibitory (MDDR)
6-Shogaol	Cyclooxygenase inhibitory (MDDR); Lipoxygenase inhibitory (MDDR)
beta-Sitosterinum (beta-Sitosterol)	Hypolipidemic (CMC)
Stigmasterin (Stigmasterol)	Antiatherosclerotic (CMC)

## Conclusions

Since only a small part of natural medicinal components have been documented in medicinal databases, the presently identified vegetable and legume functional components are only the tip of the iceberg. It is expected that with the progress of medicinal chemistry and pharmacology, more and more antidiabetic agents will be identified from foods. In fact, in a very recent study, it was reported that some triterpenoids derived from bitter melon are promising antidiabetic agents [19]. 

In nutrition studies, it is always a challenge to pinpoint the functions of food components to elucidate the epidemiological discoveries. The present study indicates that it is possible to explain (although partially) the health benefits of foods from the activities annotated in medicinal databases, which is of great significance to the study of food science and technology and even drug discovery. 

## References

[1] Tulp M., Bruhn J. G., Bohlin L. (2006). Food for thought. Drug Discov. Today.

[2] Zhang H.-Y. (2007). Can food-derived multipotent agents reduce the risk of Alzheimer's disease?. Trends Food Sci. Technol..

[3] Trichopoulou A., Costacou T., Bamia C., Trichopoulos D. (2003). Adherence to a Mediterranean diet and survival in a Greek population. New Engl. J. Med..

[4] Scarmeas N., Stern Y., Tang M. X., Mayeux R., Luchsinger J. A. (2006). Mediterranean diet and risk for Alzheimer's disease. Ann. Neurol..

[5] Yang D.-P., Kong D.-X., Zhang H.-Y. (2007). Multiple pharmacological effects of olive oil phenols. Food Chem..

[6] Fujiki H., Suganuma M., Imai K., Nakachi K. (2002). Green tea: Cancer preventive beverage and/or drug. Cancer Lett..

[7] Chen L., Zhang H.-Y. (2007). Cancer preventive mechanisms of the green tea polyphenol (-)-epigallocatechin-3-gallate. Molecules.

[8] Kuriyama S., Hozawa A., Ohmori K., Shimazu T., Matsui T., Ebihara S., Awata S., Nagatomi R., Arai H., Tsuji I. (2006). Green tea consumption and cognitive function: a cross-sectional study from the Tsurugaya Project. Am. J. Clin. Nutr..

[9] Villegas R., Shu X.-O., Gao Y.-T., Yang G., Elasy T., Li H.-L., Zheng W.  (2008). Vegetable but not fruit consumption reduces the risk of type 2 diabetes in Chinese women. J. Nutr..

[10] Villegas R., Gao Y. –T., Yang G., Li H.-L., Elasy T. A., Zheng W., Shu X.-O. (2008). Legume and soy food intake and the incidence of type 2 diabetes in the Shanghai Women’s Health Study. Am. J. Clin. Nutr..

[11] (2005). Traditional Chinese Medicine Database *(TCMD)*.

[12] Cheng A. Y., Fantus I. G. (2005). Oral antihyperglycemic therapy for type 2 diabetes mellitus. Can. Med. Assoc. J..

[13] Li J., Tian H. M., Tong N. W. (2007). Progress in drug therapy for diabetic retinopathy. West China Med. J..

[14] Luo J., Chuang T., Cheung J., Quan J., Tsai J., Sullivan C., Hector R. F., Reed M. J., Meszaros K., King S. R., Carlson T. J., Reaven G. M. (1998). Masoprocol (nordihydroguaiaretic acid): a new antihyperglycemic agent isolated from the creosote bush (*Larrea tridentata*). Eur. J. Pharmacol..

[15] Magee M. F., Taiwo A. A., Howard B. V. (2003). Management of diabetes with coronary artery disease. Curr. Treat. Opt. Cardiovasc. Med..

[16] Škrha J. (2007). Diabetes and vascular disease: From pathogenesis to treatment: Are vascular effects of hypoglycemic and hypolipidemic drugs independent of their metabolic effects?. Diabetes Metabol. Synd.: Clin. Res. Rev..

[17] (2004). Comprehensive Medicinal Chemistry *(CMC)*.

[18] (2004). MDL Drug Data Report *(MDDR)*.

[19] Tan M.-J., Ye J.-M., Turner N., Hohnen-Behrens C., Ke C.-Q., Tang C.-P., Chen T., Weiss H.-C., Gesing E.-R., Rowland A., James D. E., Ye Y. (2008). Antidiabetic activities of triterpenoids isolated from bitter melon associated with activation of the AMPK pathway. Chem. Biol..

